# Influence of Avatar Identification on the Attraction of Virtual Reality Games: Survey Study

**DOI:** 10.2196/56704

**Published:** 2024-10-22

**Authors:** PengFei Li, Fa Qi, Zhihai Ye

**Affiliations:** 1 Art College Jinan University Guangzhou China; 2 Guangzhou Node Information Techenology Co., Ltd Guangzhou China; 3 Academy of Cultural Heritage and Creativity Jinan University Guangzhou China

**Keywords:** avatar identification, self-differentiation theory, immersion, attractiveness, virtual reality games

## Abstract

**Background:**

In gaming, the embodied interaction experience of avatars serves as a key to emotional sublimation in artistic creation. This presents the emotional expression of art in a more vivid form, which is a critical factor in the high attractiveness of virtual reality (VR) games to players. Intertwined with players’ physiological and psychological responses, immersion is an essential element for enhancing gaming attractiveness.

**Objective:**

This study aims to explore how to help players establish a sense of identity with their embodied avatars in VR game environments and enhance the attractiveness of games to players through the mediating effect of immersion.

**Methods:**

We conducted a structured questionnaire survey refined through repeated validation. A total of 402 VR users were publicly recruited through the internet from March 22, 2024, to April 13, 2024. Statistical analysis was conducted using the SPSS and Amos tools, including correlation analysis, regression analysis, and mediation effect verification. We divided the self-differentiation theory into 4 dimensions to validate their impact on avatar identification. Subsequently, we correlated the effects of avatar identification, game immersion, and game attractiveness and proposed a hypothetical mediating model.

**Results:**

Regression analysis of the predictor variables and the dependent variable indicated a significant positive predictive effect (*P*<.001); the variance inflation factor values for each independent variable were all <5. In the hypothesis testing of the mediating effect, the total mediating effect was significant (*P*<.001). Regarding the direct impact, both the effect of avatar identification on immersion and the effect of immersion on game attractiveness were significant (*P*<.001). However, the direct effect of avatar identification on game attractiveness was not significant (*P*=.28). Regarding the indirect impact, the effect of avatar identification on game attractiveness was significant (*P*<.001). The results indicate a significant positive correlation between different dimensions of the self-differentiation theory and identification with avatars. Moreover, immersion in the game fully mediated the relationship between identification with avatars and game attractiveness.

**Conclusions:**

This study underscores that the embodiment of avatar identification is influenced by dimensions of self-differentiation, and the impact of identification with avatars on game attractiveness is contingent upon full mediation by immersion. These findings deepen our understanding of the role of avatar identification in VR gaming.

## Introduction

### Background

Research on avatar identification unfolds against the backdrop of the flourishing virtual reality (VR) digital gaming industry, where the allure for players is steadily increasing. According to the market research company Newzoo, the global web-based gaming user base reached 2.88 billion in 2023. Research by International Data Corporation Games indicates rapid VR and augmented reality market growth, with global expenditures projected to reach US $288 billion by 2024. This shows that gaming has become an indispensable part of people’s lives; the VR industry is thriving and poised to be integrated with gaming as a rapidly developing sector.

According to a report by Hootsuite and We Are Social, the global social media user count surpassed 4.8 billion in 2023, with nearly 60% of the world population using social media. This highlights the indispensability of digital identity in online socialization and digital communication. Aligning with the trends, an increasing number of game developers are dedicated to providing personalized and customized gaming experiences to attract players. This includes enabling players to establish unique in-game virtual identities and fostering closer connections with the game world and characters [1].

A self-avatar can replicate the user’s body posture and motions using body-tracking systems [2]. This self-avatar is experienced from a first-person perspective and, within the VR, provides a substitute body for the participant. An embodiment illusion is experienced when the participant feels the illusion that the colocated self-avatar has effectively replaced their body at a physical and functional level while immersed in the interactive virtual environment [3]. In the future metaverse era, the effective management of human digital identities will become a significant issue for governments, raising concerns regarding network information security and information disorder. If, with the support of technologies such as iris recognition, facial recognition, and motion capture, avatars align perfectly with actual individuals in terms of expressions, body language, and gestures, the merging of human and virtual identities will be achieved. When choosing avatars, individuals consider various factors, such as their authentic selves, ideal selves, preferred intellectual property images, and the social relationships formed within the virtual environment.

In the evolving landscape of VR experiences, the issue of game immersion has become a key target for academic research. Understanding how to foster resonance between individuals and their virtual characters in the narrative environment of games and leveraging the immersive experiences derived from this connection to enhance the attractiveness of games to players is a captivating proposition.

The emergence of complex virtual environments and online gaming platforms has given rise to new human-computer interaction dimensions in recent years. Individuals now have the opportunity to create avatars and play roles in the in-game space, and these avatars and roles may differ significantly from their authentic selves. This phenomenon raises intriguing questions about avatars’ psychological and physiological experiences in gaming environments. Researchers have investigated various factors related to the relationship between players and avatars, from customization to similarity [4-6]. As an emerging and crucial concept in VR games, avatar identification can be broken down into dimensions such as real self similarity, the disparity between the authentic and ideal selves, the ideal self, and social behavior. This facilitates analysis of the relationship between the self and avatar identification.

This study on avatar identification and game behavior was grounded in self-differentiation theory, focusing on the impact of avatar identification on individuals’ self-concept and self-construction. In addition, it used the stimulus-organism-response (SOR) theoretical model combined with immersion theory to investigate how avatar identification influences players’ willingness to consume virtual content to derive enjoyment and experience fun and immersion.

Exploration of the relationship between avatar identification and game immersion is critical. As individuals invest time and emotions in virtual spaces, it becomes crucial to examine how this process affects their sense of immersion in the digital gaming environment. The immersive nature of gaming experiences involves aspects such as emotional engagement, perceptual involvement, and behavioral commitment. Understanding how avatar identification aligns or misaligns with these aspects of game immersion not only contributes to the field of game psychology but also enhances our understanding of how game content creation in the VR era can increase the attractiveness of avatars to players, further enriching game narratives.

This research investigated the correlation between avatar dimensions (real self similarity, the disparity between the actual and ideal selves, the ideal self, and social behavior) and embodied avatar identification in VR games. It also explored the impact of the coordination between individuals’ authentic selves and virtual selves on the attractiveness of games and, by extension, consumption willingness. In addition, this study examined the mediating roles of emotional, perceptual, and behavioral aspects of in-game immersion.

### Theoretical Foundation and Literature Review

#### Overview

Building on the definitions of traditional media role identification and the distinctive relationship between players and in-game avatars, van Looy et al [6] proposed a 3D theoretical framework for avatar identification. This theory comprises 3 main factors, as shown in Table 1.

**Table 1:** The 3D theoretical framework for avatar identification.

Concept	Definition	Impact
Similarity identification	Involves an individual’s identification with their authentic self. People tend to appreciate and identify with fictional characters who share similarities with themselves.	Facilitates a psychological fusion between the individual and the virtual character, creating a deeper emotional connection.
Wishful identification	Involves an individual’s identification with an idealized avatar. Individuals tend to identify more with fictional characters possessing idealized traits, where such characters surpass the attractiveness of similar virtual characters.	Promotes more profound emotional connections between players and virtual characters possessing idealized traits.
Embodied presence	Reflects the psychological connection between an individual and a virtual character. It refers to sensory and behavioral manifestations of an embodiment of the virtual character and the individual’s degree of autonomy and perceptual involvement with the avatar.	Facilitates the integration of the virtual character with the individual’s authentic self, enhancing in-game immersion and fostering a more intense psychological connection.

#### Identification With Real Self Similarity

Castells [7] pointed out that self-identity is the source from which individuals derive meaning in their lives, consistent with status, interests, and belonging. According to van Bavel and Packer [8], an individual’s preferences are influenced by social identity, where, to some extent, social identity represents who one is as a person. When social identity scratches the *itch* of *needs*, people attach great importance to social groups. People value 3 primary psychological needs: belonging, uniqueness, and status [8]. Belonging arises from identity, ethnicity, culture, environment, and social groups, simultaneous with exclusivity. Uniqueness arises from individual comparisons within the group, manifesting as self-identification and the identification of others with oneself. Status, which refers to the individual’s position and rank within the group, involves identity recognition and psychological compensation. These 3 interdependent needs provide a basis for identity recognition in the real world.

In the gaming context, an individual’s identification with the avatar arises from both subjective and objective self-recognition, including physiological and psychological recognition as well as recognition by others with whom one has social relationships.

#### Understanding the Discrepancy Between the Real and Ideal Selves

According to self-discrepancy theory [9], an individual’s actual self represents their self-perceived characteristics and those others perceive. On the other hand, the ideal self represents the traits one or others wish the individual possessed. Real–ideal self discrepancy is the perception of the individual that their actual self falls short of the perfect self, leading to depressive emotions such as disappointment and frustration. To mitigate the negative emotional experiences caused by the real–ideal self discrepancy, individuals often resort to maladaptive coping strategies, resulting in psychological and behavioral issues. Games can, to a certain extent, help individuals emotionally compensate in situations involving emotional setbacks.

Scholars have explored game motivations and found that many players focus on pleasure and social motivations. Wei et al [10] identified pleasure as the most critical aspect of player motivation. Xie and Zhang [11] proposed that intrinsic motivations (enjoyment, self-efficacy, social interaction, and transcending reality) and extrinsic motivations, which collectively influence online game players’ gaming intentions and behavior. They also emphasized the interaction between intrinsic and extrinsic motivations. In games, avatars can fulfill players’ desires regarding personal achievement, self-value realization, virtual social interactions, and transcending the self. Therefore, players are motivated to achieve idealized identification with their avatars.

#### Individual Identification With the Ideal Self Represented by Avatars

An avatar is the in-game representation of the self actively manipulated in the virtual world, serving as a tangible connection point between the virtual and real worlds. In gaming, avatars are virtual self-representations that players can see and control in real time within the virtual environment [12]. By using and experiencing game avatars, a psychological phenomenon known as idealized avatar identification emerges. This phenomenon involves individuals integrating with their avatars, enhancing cognitive and emotional connections, adopting a positive attitude toward avatars, and experiencing a lack of self-awareness. The extent of identification is based on the similarity between avatars and individuals, the attractiveness of individuals, and the degree to which individual needs are satisfied [13].

According to the perspective of symbolic interactionism on the self, individuals develop various identities throughout their lives, such as social, personal, and role identities [14]. Idealized avatar identification is described as an individual’s “expectancy cognitive linkage” [15], a “meaningful positive psychological connection” with avatars [16], or an “attachment” [17]. Idealized avatars allow individuals to construct subjective images of themselves and their social relationships, achieve psychological satisfaction, and meet goals through gaming.

#### Individual Identification With the Social Behaviors of Avatars

The looking-glass self theory by Cooley [18] posits that self-perception heavily influences human behavior, primarily through social interactions with others. Others’ evaluations, attitudes, and reflections serve as a “mirror” reflecting the self, allowing individuals to understand and view themselves through this mirror. Social media users transmit and exchange information through self-avatars. On the one hand, this process helps delineate avatars’ ideal appearance and identity more clearly. On the other hand, it continuously refines understanding one’s true self. Other avatars synchronously evaluate the true self and the avatar and, through self-observation, infer their attitudes and beliefs and manage cognitive and emotional states. We define user avatar identity as the degree to which users expand their identity [15,19,20]. The relationship between users and avatars is a merged identity [6].

#### Impact of Avatar Identification on Game Immersion

Drawing on the characteristics of games, Klimmt et al [13] proposed the theory of player-avatar identification based on the identity construction theory. Avatar identification is a social-psychological phenomenon related to media users’ self-perceptions and identification. A crucial feature of avatar identification is the transfer of player self-perception, where players merge with in-game avatars, temporarily treating themselves as game characters. The game avatar, as a form of virtual self-presentation, is internalized by players and influences self-identity. The most prominent features of VR are interactivity, immersion, and imagination bind the player’s body closely to the character [21], creating a harmonious unity between humans and machines. Emotional, perceptual, and behavioral immersion in games, combined with avatar identification, build a strong bond with the avatar crafted in the game environment.

In the visually immersive scenes created by VR, users need to master basic abilities such as object recognition and motor skills. This participatory experience is a process with exploratory and skill-based elements. It involves the perception of information through the visual nervous system, engages other organs through visual system stimulation, and requires mental energy investment, leading to the experience of avatar identification and flow in the VR environment.

#### Role of Avatar Identification in the Attractiveness of VR Games and the Mediating Effect of Immersion

Csikszentmihalyi [22] introduced the concept of flow as an emotional experience where individuals exhibit a strong interest in an activity, prompting complete engagement. Brown and Cairns [23] proposed 3 stages of flow: engagement, concentration, and full immersion. Players become involved in the game by understanding its narrative and gameplay. Familiarity with the characters promotes interest based on the similarity to the authentic self, fostering empathy with the characters. Players who complete tasks and collaborate gain confidence and cooperative skills, achieving ideal self identification and social behavior identification with the in-game characters. This process, progressing from concentration to complete immersion, generates emotional attachment.

Compared to traditional games, VR games offer a perfect immersive, interactive experience regarding players’ visual and auditory sensations. The sense of identification with avatars is novel and involves similarity identification, where players immerse themselves in the game and integrate themselves with their characters. Psychologically, players experience emotional changes through an idealized avatar. At a behavioral level, players have simulated interactive experiences, becoming “as one” with their avatars. Player motivation and narrative interactions with nonplayer characters (NPCs) and other players enhance the embodiment effect, making the game highly attractive to players and influencing their willingness to consume in-game content.

On the basis of the aforementioned research, several research questions can be derived: (1) does self similarity (ie, the difference between the authentic and ideal selves, the ideal self, and social behavior) determine “players’” identification with avatars? (2) Does players’ identification with avatars affect immersion? and (3) Does immersion mediate the relationship between avatar identification and the game’s attractiveness to players?

### Research Hypotheses

#### Hypothesized Model of Avatar Identification

Avatar identification relies on actual self similarity, differences between the actual and ideal selves, identification with the perfect self, and identification with social behavior. Actual self similarity identification subsumes aspects of identity such as gender, age, and psychological belongingness to the collective and environmental space. Identity is at the core of an individual’s social position, including its maintenance and enhancement [24]. Individuals seek self-affirmation and group identity through collective survival as social beings, such as through labor identity related to productive work and identity in social interactions [25]. Therefore, in identifying with actual self similarity, individuals choose avatars with features similar to those of their actual selves to achieve identity. Age, gender, and other appearance-related characteristics are basic distinguishing features of individuals in social groups and provide a foundation for self-identification in association with the real world. The social groupness of individuals is not only based on external appearance but also relies on participation and self-control in social activities to position oneself in the group and achieve value recognition.

Without “human emotions,” truth cannot be pursued. Genuine identification involves warmth and emotional connection. Without human emotions, there can be no genuine identification [26]. Therefore, psychological and emotional factors are crucial for authentic identification. Without emotions, it is impossible to identify with the actual self. Avatar identification subsumes sensory aspects and rational emotions. In the individual-team relationship dynamic, individuals emotionally invest in organizations they trust and, at the same time, evaluate the group environment and their values rationally. Abilities, personality traits, cognitions, emotions, and emotional values determine one’s identification with a group and, in turn, influence the individual’s evaluation of their value contribution to that group. Given these considerations, we propose the following hypothesis: positive self similarity identification with the actual self positively impacts avatar identification (hypothesis 1.1).

The disparity between the actual and ideal selves positively influences avatar identification, providing intrinsic and extrinsic motivation for players. This includes individual achievements, self-worth realization, virtual social interactions, and transcending one’s authentic self. In the context of Western philosophical traditions, personal autonomy and authenticity concepts have been focal points in the debate between communitarianism and liberalism. Autonomy emphasizes an individual’s capacity for self-restraint and self-management according to social orders and rules. At the same time, pursuing authenticity or one’s true self reflects the ideal of remaining faithful to one’s inner calling, an important topic of discussion in contemporary society. According to self-discrepancy theory [9], ideal self identification is the willingness to realize self-desires about oneself and others. Player motivation involves recognizing autonomous thoughts, where each player desires to plan and achieves their own vision of a good life. Honneth [27] argues that only through the achievement of self-confidence, self-esteem, and self-respect “...can a person unconditionally regard himself as an independent individual, identifying with his or her goals and ideals.” In virtual games, players, based on game settings, gain a sense of achievement when completing tasks and receive assistance within virtual gaming communities. This transition from self-value realization to psychological transcendence of the self is facilitated through the construction of virtual world rule consciousness using avatars. Given these considerations, we propose the following hypothesis: disparities between the actual and ideal selves positively influence avatar identification (hypothesis 1.2).

Ideal self identification influences avatar identification positively. Ideal self identification manifests as positive psychological expressions based on goal and cultural identification. Goal identification relates to the confidence gained from completing tasks, obtaining high-visibility honors, and receiving differentiated services. Cultural identification arises from cognitive perspectives of narrative content; subjective preferences for certain types of content; and investment of money, time, and emotional attachment. Kellner [28] argues that the media provides potent images and scenes with which one can identify, which can directly influence people’s behavior while also offering models for action, fashion, and style. The media plays a crucial role in forming individual self-identity and national identity. Media such as movies and games significantly influence the formation of viewers' and players' thoughts, beliefs, and values, assuming a dominant role in shaping national cultural identity [29]. Constructing a subjective cultural identity encourages individuals to identify with specific characters, images, or positions. For example, resonating with information about the game’s narrative content corrects individual cognitive biases, generating affection and familiarity with characters, relationships, and environmental scenes within the narrative framework, thereby promoting idealized identification. The phenomenon of identification with the culture among young people is evident. “Secondary creations,” such as the role-playing of characters, fan art, and pairing the self with in-game characters, involve personal, idealized reprocessing of emotions. Attitudes toward narrative plots, cultural scenes, environments, and artistic style expressions and the level of affection for individual characters determine player attitudes. Given these considerations, we propose the following hypothesis: ideal self identification influences avatar identification positively (hypothesis 1.3).

Social behavior identification influences avatar identification positively. Social behavior identification encompasses player motivations and social behaviors toward the self, NPCs, player groups, and game product creators. Game creators aim to create cultural, community, and intangible assets related to game content within the game’s narrative space, guiding players to actively participate in building the game ecosystem and content creation. Players’ value judgments and achievements determine their social and behavioral identification with the group. This is manifested in the player’s degree of attachment to the game, which influences the investment of money, time, and emotions. The “dramaturgical theory” by Goffman [30] suggests a high degree of interaction and strong correlation among media, scenes, and behaviors. Space and environment, real-time individual status, individual living habits, and social atmosphere are considered the 5 essential elements of a scene. When users enter the game space from the real world, they must first change their identity status and “play” a virtual role to achieve psychological and physiological unity (especially in interactive VR simulation games with a visual experience from a first-person perspective). Players’ social interactions may involve familiar or unfamiliar characters during the game, but everyone collaborates to fulfill common game objectives. This indicates a consensus regarding values, and no player perceives the shared fictional plot as boring or meaningless. Through in-game cooperation, players quickly develop empathy and even friendships. When players act out their roles, they identify with their character’s identity, NPCs, and the player community. Everyone derives shared values from that place, whether the game is cooperative or competitive. Given these considerations, we propose the following hypothesis: social behavior identification influences avatar identification positively (hypothesis 1.4).

**Figure 1:**
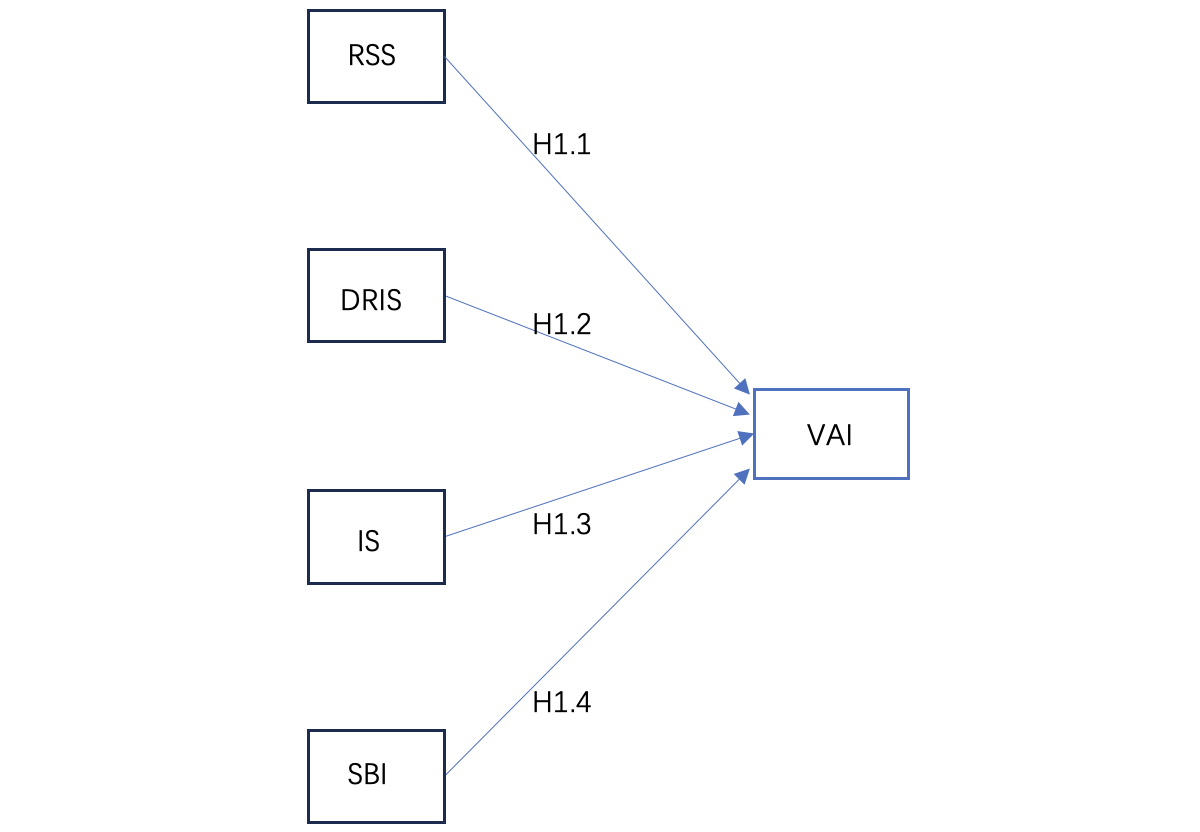
The hypothesis framework of 4 dimensions and incarnation identity—avatar identification hypothesis model. DRIS: discrepancy between the real and ideal selves; H: hypothesis; IS: ideal self; RSS: real self similarity; SBI: social behavioral identity; VAI: virtual avatar identity.

On the basis of the aforementioned discussion, a hypothesis model of avatar identification was designed, as shown in Figure 1.

#### Mediation Hypothesis Model

##### The SOR Theory

As a significant paradigm in psychology, the SOR theoretical model is an optimized model derived by Mehrabian and Russell [31] based on the perspective of environmental psychology. It integrates the organism into the original stimulus response model. This model assumes that information cues in the external environment induce cognitive or emotional changes in the organism in response to stimuli, leading to approach behavior toward positive stimuli or avoidance of negative stimuli.

**Figure 2:**
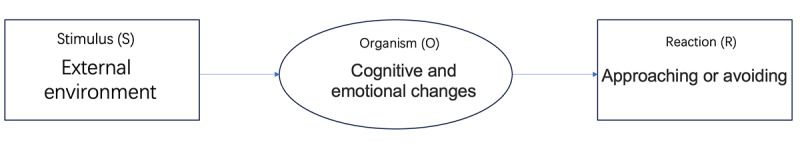
Psychological model of the theoretical stimulus-organism-reaction model.

In this model, stimuli are information cues present in the collective environment, and the organism represents the individual’s internal cognitive and emotional activities in response to external stimuli. By influencing the individual’s cognitions and emotions, these external environmental factors further affect the individual’s responses, including attitudes and behavior (approach or avoidance). Suppose external ecological factors are experienced as positive stimuli. In that case, they trigger approach behaviors, and the resulting response may involve staying still, further exploring, or developing an emotional attachment to the environment. Conversely, stimuli experienced as unfavorable induce avoidance behaviors, leading to opposing and evasive responses, as illustrated in Figure 2.

Currently, the SOR model is widely applied in consumer behavior. Wang et al [32] applied the SOR model to study the live-streaming e-commerce model. Their research conceptualized the interactivity, entertainment, and promotional aspects of the live-streaming e-commerce environment as external environmental stimuli. The emotional and cognitive states of live stream audiences were considered the organism, and the study focused on examining consumer attitudes and behaviors in the context of live streaming.

Oldenburg [33] posited that online games create a “third place” for players outside of the work and home environments. In this virtual world, players create and control avatars, collaborate with other players, achieve goals, and establish a network of relationships that bridge the online and offline worlds and the in-game and out-of-game experiences.

In VR gaming, the fit between players and avatars is better than in traditional online games, offering advantages regarding player presence, social activities, simulated interactions, and embodiment. Therefore, the influence of external stimuli on internal emotional changes as per the SOR model is relevant. Using the SOR model as a theoretical reference for this study was deemed appropriate.

##### Design of the Mediation Hypothesis Model

This study explored the relationship between avatar identification and player immersion, subsequently influencing the pathway through which VR game content attracts players. This study adopted the SOR psychological paradigm as the foundational model considering the characteristics of VR games. Immersion was selected as the mediating variable to construct a model illustrating the impact of avatar identification on the attractiveness of VR games.

**Figure 3:**
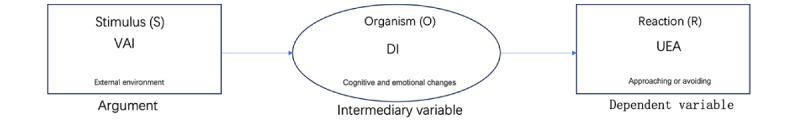
The avatar identification–immersion–game attractiveness model based on the stimulus-organism-reaction theory. DI: immersion; UEA: user experience attractiveness; VAI: virtual avatar identity.

In this model, avatar identification is considered an external environmental stimulus (S), player attraction to VR games is considered to depend on the attitudinal and behavioral aspects of the approach response (R), and player immersion in the gaming experience (perceptual, emotional, and behavioral immersion) is regarded as a reflection of cognitive and emotional changes in the organism (O). The conceptual model was constructed with avatar identification as the independent variable, player attraction to VR games as the dependent variable, and immersion as the mediating variable, as illustrated in Figure 3.

##### Immersion as the Mediating Variable

Identification with the real self, the difference between the authentic and ideal selves, ideal self identification, and social behavior identification are the constituents of avatar identification. Merleau-Ponty and Smith [34] conceptualize human existence in terms of the subjective experience of the body. They view the body as “a natural me and a perceptual subject,” signifying that the body is not merely physically present but also serves as a “perceptual body” with holistic attributes of perception, emotion, and behavior. Hence, the immersive experience engendered by VR games comprises perceptual, emotional, and behavioral immersion.

Research suggests that the human mind and behavior are context sensitive, where environmental factors, including technology, may unconsciously influence players [35]. In immersive interactive environments, technology, the environment, and the body achieve a high degree of integration, affecting cognition, behavior, and life in general [36]. The immersive interactive scenes in VR games break the sense of an “out-of-place” body in nonimmersive communication modalities, creating a sense of embodiment within the avatar [37].

The elements of authentic self, ideal self, and social behavior identification with the avatar correspond to perceptual, emotional, and behavioral immersion, respectively. These aspects reflect the embodied experience, psychological feelings, and interactive behaviors. The difference between the authentic and ideal selves reflects player motivation. Norman [38] proposed that the human emotional system operates on 3 levels: instinct, behavior, and reflection. The instinct level includes a series of unconscious experiences dominated by the sensory system, the behavioral level involves interaction with the product to fulfill functional needs, and the reflective level emphasizes the inner thoughts and emotional satisfaction that users gain during the game experience [38]. Game immersion begins with sensory perception, emotional, psychological, and behavioral experiences. Given these considerations, we propose the following hypothesis: avatar identification has a positive impact on game immersion (hypothesis 2).

##### Game Attractiveness as the Dependent Variable

Immersion is a profound user experience characterized by complete involvement in an activity that leads to a self-forgetful state, resulting in fulfillment and happiness [39]. Embodied emotional experiences are a critical factor that allows VR games to attract players strongly. Players gain a sense of achievement and experience entertainment during the gaming process, leading to joyful experiences and happiness arising from the release of dopamine (a reward neurotransmitter) in the brain. Through the unique characteristics of VR games, players are provided with a fully immersive and embodied virtual experience involving visual, auditory, and behavioral simulated interactions, which is crucial for the attractiveness of games to players. Designers evoke sensory perceptions through scene and space manipulation to capture players’ attention. Avatar identification achieves emotional resonance between the game character and the player. Finally, at the level of behavior, interactions between players and in-game characters impact players’ willingness to engage with the game. Given these considerations, we propose the following hypotheses: immersion mediates the relationship between avatar identification and game attractiveness to players (hypothesis 3), and avatar identification positively influences game attractiveness (hypothesis 4).

**Figure 4:**
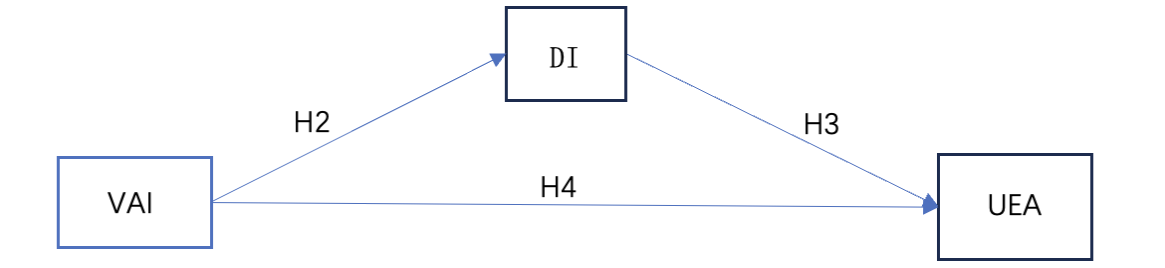
The avatar identification–immersion–game attractiveness mediation hypothesis model. DI: immersion; GA: game attractiveness; H: hypothesis; VAI: virtual avatar identity.

The mediation hypothesis model designed in this study is shown in Figure 4.

### Academic Contribution

Focusing on the development of the VR industry, this research delves into the study of avatar identification and game attractiveness. Existing studies belong to the fields of gaming, psychology, and sociology. This research emphasizes avatar identification, a critical issue for the impending era of the metaverse, where humans transition from real-world identities to 3D virtual spaces, inevitably facing challenges related to avatar identification. Avatar identification involves physiological and psychological aspects, addressing issues of identification and acceptance of the virtual self and legal considerations regarding identity ID.

Therefore, the study of avatar identification is of great importance. It helps understand how individuals relate to their virtual selves and raises broader questions about identity in digital spaces. In addition, this research is closely related to immersion and game attractiveness. Analyzing the mediating role of immersion and the consumption intentions of game users can provide valuable insights and professional guidance for the industry’s development. This research contributes to understanding how avatar identification impacts player engagement and behavior in virtual environments, which is crucial for designing more immersive and appealing VR experiences.

## Methods

### Design and Study Population

We conducted a survey targeting VR game users on forums with high discussion activity related to VR gaming platforms, such as Sony PlayStation VR, PICO, SteamVR, and Quest. In addition, we extended invitations on more widely used platforms such as YouTube and Instagram, as well as VR forums in mainland China and Taiwan. Participants accessed the questionnaire via a link, and a written informed consent form was placed at the top of the questionnaire page. This study used a questionnaire consisting of 7 demographic questions and 37 survey questions on a 5-point Likert scale. Participants were required to first read the “Letter to Survey Participants” and the “Informed Consent Form” to understand the basic details of the study, the risks involved, and their rights and confirm their consent to participate in the survey.

We conducted a questionnaire survey using a simple random cluster sampling method, targeting individuals with experience in VR gaming. An electronic questionnaire was developed using Wenjuanxing, and the online poll lasted for 1 month. A total of 402 VR users were publicly recruited through the internet from March 22, 2024, to April 13, 2024. All participants read the informed consent form before the start of the questionnaire, voluntarily agreed to participate in this survey, and agreed to the use of the information collected in this survey for other research purposes by the study leader or researchers outside of this study. Due to limitations in research funding, the uneven distribution of VR users, and the fact that all survey participants were adults, the sample size of this study was relatively small. The data obtained from this survey may not necessarily represent the actual needs of all users. However, based on the data from this survey, there is research significance in exploring the effects of avatar identification and immersion, which can help us gain a universal consensus on improving the attractiveness of VR games through the actual feelings of this group of people.

### Measurement Tools

The measurement scale for this study was developed based on the self-distinction theory [9] and the self-identity scale [40]. It incorporates elements from the Game Immersion Scale developed by Choi and Kim [41], the Avatar Identification Scale created by van Looy et al [16], and the Game Addiction Scale [42] for design and compilation. The scale comprises 7 dimensions: real self similarity, discrepancy between the real and ideal selves, ideal self, social behavior, avatar identification, immersion, and attractiveness of the game to players (N=37 items). A 5-point Likert scale was used for scoring the items (1=strongly disagree; 5=strongly agree), as shown in Table 2.

**Table 2:** Measurement scale (N=37 items).

Latent variable	Question items	Reference source
RSS^a^	A1—when creating game characters, my avatar is very similar to me. A2—when creating an in-game avatar, I highlight my strengths, such as physique, appearance, hairstyle, and profession. A3—when my game avatar faces danger in the game, I feel nervous. A4—in the game, when the team I represent achieves victory, I feel very happy.	Higgins [9]; Zhang [40]
DRIS^b^	B1—when I play my favorite game, I want to achieve higher levels and accomplishments. B2—when playing my favorite game, I imagine my avatar as a perfect character. B3—in games, I aspire to make more friends or help others compared to the real world. B4—in games, I exhibit a stronger sense of competitiveness or ambition compared to my real-life self.	Higgins [9]; Zhang [40]
IS^c^	C1—when I complete the in-game objectives or tasks, I feel confident or happy. C2—when I play games, I make an effort to complete the objectives and tasks set within the game. C3—I choose game characters of the same type because I like a certain culture. C4—I choose the appearance, clothing, skills, and equipment that I like for my virtual character.	Higgins [9]; Zhang [40]
SBI^d^	D1—when playing my favorite game, I care a lot about my performance in the game or sharing my gaming achievements on social media. D2—in the games I like, I actively participate in UGCe or browse related information on forums. D3—when playing my favorite multiplayer cooperative games, I actively collaborate with other players to complete game tasks even though I may not know them in the real world. D4—in my favorite game, I interact with NPCsf to gain a better understanding of the game’s storyline.	Zhang [40]; Heng et al [43]
VAI^g^	E1—it is important for me that the avatar I customize is unique and stands out from others. E2—when I use my avatar, I feel like I am my avatar. E3—my avatar possesses some characteristics that I desire to have. E4—I am satisfied with the avatar I am currently using (or have used before). E5—the props or abilities possessed by my avatar match my expectations. E6—I am satisfied with the status and identity of my in-game avatar.	van Looy et al [16]
DI^h^	F1—when playing VRi games, I feel like I go to a completely new place and immerse myself in it. F2—I feel like I am controlling an avatar when playing VR games. F3—sometimes, when I am playing my favorite game, I forget about time and the surrounding environment. F4—when playing my favorite game, I thoroughly enjoy the visual graphics and immerse myself in them. F5—when playing my favorite game, I thoroughly enjoy the music and sound effects, becoming fully immersed in them. F6—when playing VR games, I feel like I have performed the actions myself when my avatar performs actions according to my commands. F7—when playing VR games, I often feel that the in-game items I hold are real objects, not just a game controller.	Choi and Kim [41]; Yee [44]
GA^j^	G1—in the game, I am not bound by the various constraints of reality and can do whatever I want. G2—things that are impossible in real life can be done in games. G3—through in-game battles and attacks, I can release the frustrations and suppressed feelings experienced in reality. G4—whenever I am feeling down, I prefer to play games. G5—I am very interested in the virtual game world. G6—in the game, I can experience the joy of exploring the world and seeking novelty. G7—in games, I can fully enjoy the thrill of adventure and combat. G8—in the game, I receive equal or even improved treatment from others.	Young [42]

^a^RSS: real self similarity.

^b^DRIS: discrepancy between the real and ideal selves.

^c^IS: ideal self.

^d^SBI: social behavioral identity.

^e^UGC: user-generated content.

^f^NPC: nonplayer character.

^g^VAI: virtual avatar identity.

^h^DI: immersion.

^i^VR: virtual reality.

^j^GA: game attractiveness.

### Ethical Considerations

This study received an exemption approval from the institutional review board of the Social Sciences and Humanities Department of Jinan University, China (approval B2403001-036). The original informed consent form described that the information collected from the survey could be used for secondary research by researchers or research personnel other than those conducting this study. All research data were anonymous, and each survey participant was provided with a VR game worth US $4.99 as compensation. The anonymity of participants was ensured, and no individual participant could be identified from any images in the manuscript or supplementary materials.

## Results

### Data Collection

We ultimately collected 402 valid responses, with all participants being fully informed and consenting to the collection of questionnaire information. The questionnaire had a 100% (402/402) response rate, and the descriptive statistics of the survey sample are presented in Table 3.

**Table 3:** Sample descriptive statistics (N=402).

Category		Participants, n (%)
**Gender**		
	Male	238 (59.2)
	Female	164 (40.8)
	Intersex	0 (0)
**Career**		
	Student	168 (41.8)
	Civil servant	8 (2)
	Private-sector employee	76 (18.9)
	Self-employed	61 (15.2)
	Working in a medical or educational institution	36 (9)
	Other	53 (13.2)
**Average time spent gaming per week**		
	Almost no gaming	5 (1.2)
	<1 h	23 (5.7)
	1-5 h	71 (17.7)
	5-10 h	114 (28.4)
	>10 h	189 (47)
**Preferred types of multiplayer games (multiple options)**		
	Single-player games	291 (72.4)
	Cooperative (multiplayer collaboration) games	327 (81.3)
	Competitive (multiplayer battle) games	339 (84.3)
**Played VR^a^ games before**		
	Yes	402 (100)
	No	0 (0)

^a^VR: virtual reality.

Regarding gender distribution, 40.8% (164/402) of the participants were female, 59.2% (238/402) were male, and 0% (0/402) were intersex. The slightly higher proportion of male participants aligns with the general male predominance among gamers [45].

In total, 41.8% (168/402) of the participants were students, 2% (8/402) were civil servants, 18.9% (76/402) were private-sector employees, 15.2% (61/402) were self-employed, and 9% (36/402) worked in medical or educational institutions. In addition, 13.2% (53/402) of the participants had “other” occupations. These distributions reflect the high proportion of students in the gaming community.

Regarding the average time spent gaming per week, 1.2% (5/402) of the participants reported almost no gaming, 5.7% (23/402) played for <1 hour per week, 17.7% (71/402) played for 1 to 5 hours per week, 28.4% (114/402) played for 5 to 10 hours per week, and 47% (189/402) played for >10 hours per week. These data are consistent with the expected characteristics of individuals interested in gaming.

Regarding the preferred types of multiplayer games, participants could choose from among multiple options. Single-player games were favored by 72.4% (291/402) of the respondents, cooperative (multiplayer collaboration) games were preferred by 81.3% (327/402) of the individuals, and competitive (multiplayer battle) games were the games most enjoyed by the participants (339/402, 84.3%). Thus, most respondents preferred multiplayer games. All participants (402/402, 100%) had experience playing VR games.

### Analysis

The questionnaire was revised following the completion of the initial version and then subjected to 2 small-scale tests incorporating expert feedback. The SPSS software (version 27; IBM Corp) was used for reliability and validity analyses. In the reliability analysis, the Cronbach α coefficient for the overall scale was >0.8 (0.981), indicating good data reliability. As shown in Table 4, the data underwent Kaiser-Meyer-Olkin and Bartlett sphericity tests. The Kaiser-Meyer-Olkin test statistic was >0.8 (0.986), and the Bartlett test yielded a value of 8374.366 with a corresponding *P* value of <.001. This suggests that the questionnaire has excellent overall validity and supporting factor analysis, as shown in Table 4.

**Table 4:** Cronbach reliability analysis (N=402; overall Cronbach α>0.8).

Variable	Items, n	Sample size, n (%)	Cronbach α
RSS^a^	4	402 (100)	0.825
DRIS^b^	4	402 (100)	0.855
IS^c^	4	402 (100)	0.852
SBI^d^	4	402 (100)	0.863
VAI^e^	6	402 (100)	0.895
DI^f^	7	402 (100)	0.915
GA^g^	8	402 (100)	0.923

^a^RSS: real self similarity.

^b^DRIS: discrepancy between the real and ideal selves.

^c^IS: ideal self.

^d^SBI: social behavioral identity.

^e^VAI: virtual avatar identity.

^f^DI: immersion.

^g^GA: game attractiveness.

After the preliminary examination, an exploratory factor analysis was conducted to assess the questionnaire’s validity. The 37 scale items were divided into 7 dimensions. Items loaded on 2 dimensions with factor loadings of >0.5 were removed if they did not pass the validity check. The removed items were A3, B3, C3, D2, D4, F6, G1, G2, G3, and G4. The remaining items were considered valid and retained after passing the validity check, as shown in Textbox 1.

**Mapping of variables to factors (real self similarity [RSS]=3; discrepancy between the real and ideal selves [DRIS]=3; ideal self [IS]=3; social behavioral identity [SBI]=2; virtual avatar identity [VAI]=6; immersion [DI]=6; game attractiveness [GA]=4).**

**Variables and factors**

- RSS: items A1, A2, and A4
- DRIS: items B1, B2, and B4
- IS: items C1, C2, and C4
- SBI: items D2 and D4
- VAI: items E1, E2, E3, E4, E5, and E6
- DI: items F1, F2, F3, F4, F5, and F7
- GA: items G5, G6, G7, and G8

On the basis of the comprehensive analysis, the items were categorized as follows: (1) items A1, A2, and A4 belong to the real self similarity dimension; (2) items B1, B2, and B4 belong to the discrepancy between the real and ideal selves dimension; (3) items C1, C2, and C4 belong to the ideal self dimension; (4) items D2 and D4 belong to the social behavioral identity dimension; (5) items E1, E2, E3, E4, E5, and E6 belong to the avatar identity dimension; (6) items F1, F2, F3, F4, F5, and F7 belong to the immersion dimension; and (7) items G5, G6, G7, and G8 belong to the user experience attractiveness dimension.

The results indicated good structural validity of the data (Table 5).

**Table 5:** Results of exploratory factor analysis (the results indicated good structural validity of the data).

Item	Factor coefficient		Common degree (public factor difference)
	Emotional experience (F1)	Subjective perception (F2)	
A1	—^a^	0.791	0.696
A2	—	0.688	0.637
A4	0.769	—	0.706
B1	0.688	—	0.661
B2	—	0.675	0.679
B4	0.577	—	0.564
C1	0.698	—	0.667
C2	0.671	—	0.559
C4	0.672	—	0.589
D1	—	0.582	0.588
D3	0.699	—	0.662
E1	—	0.565	0.566
E2	—	0.704	0.620
E3	—	0.679	0.660
E4	—	0.649	0.665
E5	—	0.623	0.560
E6	—	0.636	0.639
F1	0.672	—	0.683
F2	0.580	—	0.574
F3	0.634	—	0.638
F4	0.712	—	0.742
F5	0.638	—	0.551
F7	0.544	—	0.542
G5	0.689	—	0.586
G6	0.677	—	0.675
G7	0.744	—	0.728
G8	0.645	—	0.564

^a^Values less than 0.5 have been omitted.

### Avatar Identification Hypothesis Model

The results of the correlation analysis are shown in Table 6.

**Table 6:** Results of the correlation analysis of the average scores for the 7 dimensions in the model: real self similarity (RSS), discrepancy between the real and ideal selves (DRIS), ideal self (IS), social behavioral identity (SBI), avatar identity (VAI), immersion (DI), and game attractiveness (GA; N=402).

		RSS	DRIS	IS	SBI	VAI	DI	GA
**RSS**								
	*r*	1	0.802	0.788	0.771	0.83	0.844	0.801
	*P* value	—^a^	<.001	<.001	<.001	<.001	<.001	<.001
**DRIS**								
	*r*	0.802	1	0.799	0.789	0.836	0.845	0.835
	*P* value	<.001	—	<.001	<.001	<.001	<.001	<.001
**IS**								
	*r*	0.788	0.799	1	0.78	0.805	0.846	0.827
	*P* value	<.001	<.001	—	<.001	<.001	<.001	<.001
**SBI**								
	*r*	0.771	0.789	0.780	1	0.807	0.817	0.798
	*P* value	<.001	<.001	<.001	—	<.001	<.001	<.001
**VAI**								
	*r*	0.830	0.836	0.805	0.807	1	0.867	0.831
	*P* value	<.001	<.001	<.001	<.001	—	<.001	<.001
**DI**								
	*r*	0.844	0.845	0.846	0.817	0.867	1	0.885
	*P* value	<.001	<.001	<.001	<.001	<.001	—	<.001
**GA**								
	*r*	0.801	0.835	0.827	0.798	0.831	0.885	1
	*P* value	<.001	<.001	<.001	<.001	<.001	<.001	—

^a^Not applicable.

Regression analysis of the predictor variables (real self similarity, disparity between the actual and ideal selves, ideal self, and social behavior) and the dependent variable (virtual identity) indicated a significant positive predictive effect; the variance inflation factor values for each independent variable were all <5. Real self similarity was statistical significance positively correlated with virtual identity (β=.277; *P*<.001), the disparity between the actual and ideal selves was statistical significance positively correlated with virtual identity (β=.258; *P*<.001), the ideal self was statistical significance positively correlated with virtual identity (β=.161; *P*<.001), and social behavior was statistical significance positively correlated with virtual embodiment identity (β=.192; *P*<.001), as shown in Table 7.

**Figure 5:**
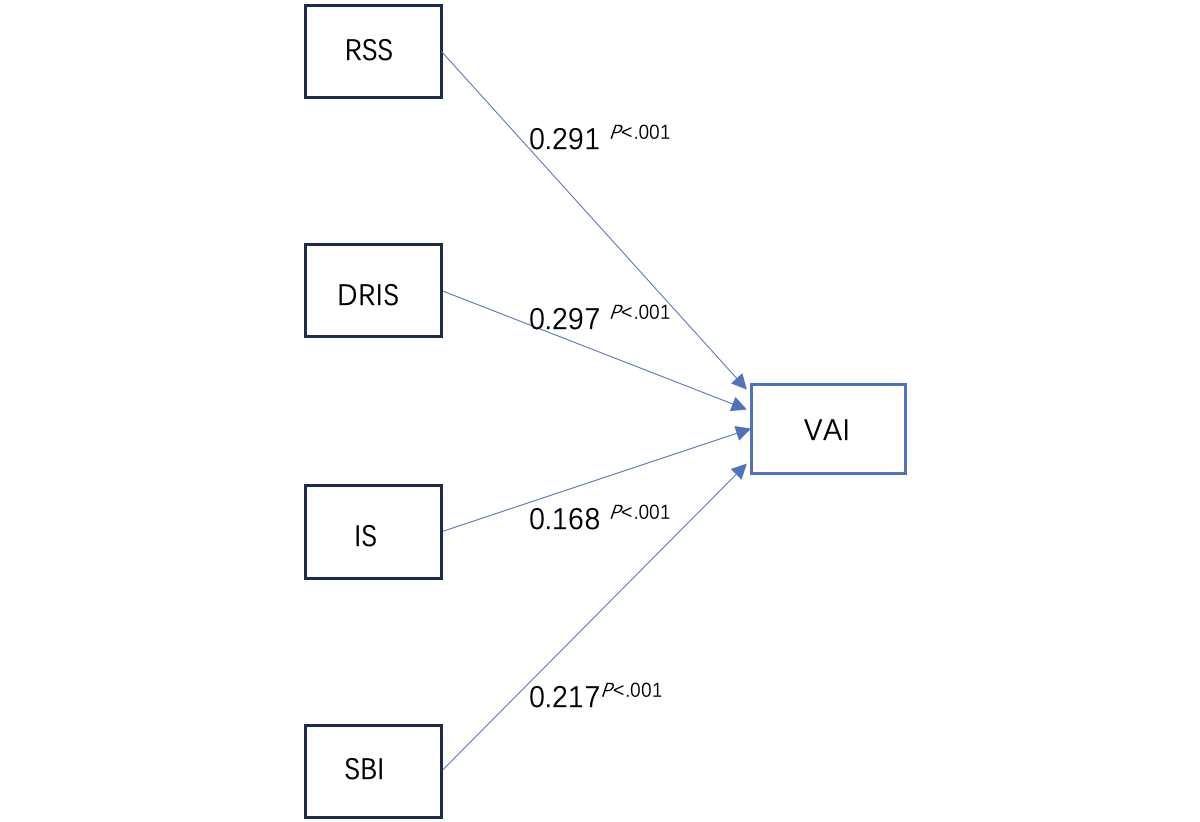
Results of the validation of the avatar identification model (P<.001). DRIS: discrepancy between the real and ideal selves; IS: ideal self; RSS: real self similarity; SBI: social behavioral identity; VAI: virtual avatar identity.

On the basis of the hypothesis-testing analysis of the model, the avatar identification hypothesis model was derived, as shown in Figure 5. Real self similarity, the disparity between the actual and ideal selves, the ideal self, and social behavior statistical significance positively affect avatar identification.

**Table 7:** Results of regression analysis of identity variables related to avatars (dependent variable: virtual avatar identity; variance inflation factor>5; *P*<.001).

Model	Unstandardized coefficients, B (SE; 95% CI)	Standardized coefficients, β	*t* test (*df*)	*P* value	Collinearity statistics	
					Tolerance	Variance inflation factor
Constant	0.385 (0.083; 0.222-0.547)	—^a^	4.651 (401)	<.001	—	—
RSS^b^	0.277 (0.040; 0.198-0.357)	.291	6.857 (401)	<.001	0.278	3.603
DRIS^c^	0.258 (0.038; 0.183-0.334)	.297	6.718 (401)	<.001	0.256	3.903
IS^d^	0.161 (0.041; 0.081-0.241)	.168	3.937 (401)	<.001	0.274	3.646
SBI^e^	0.192 (0.037; 0.120-0.264)	.217	5.265 (401)	<.001	0.295	3.387

^a^Not applicable.

^b^RSS: real self similarity.

^c^DRIS: discrepancy between the real and ideal selves.

^d^IS: ideal self.

^e^SBI: social behavioral identity.

### Mediation Effect Analysis

The results of the correlation analysis indicated that the relationships among avatar identification, immersion, and the game’s attractiveness to players satisfied the conditions for conducting a mediation effect test. Using the SPSS (version 27) and SPSS Amos (version 25; IBM Corp) software, the independent variable of avatar identification, the dependent variable of attractiveness, and the mediating variable of immersion were tested sequentially, and the mediation effect was assessed by estimating its 95% CI. A CI not containing 0 indicates statistical significance. The model fit indexes were as follows: *χ*^2^_101_=104.8, Incremental Fit Index=0.999 (>0.9), Comparative Fit Index=0.969 (>0.9), Tucker-Lewis Index=0.999 (>0.9), and *P*=.38 (>.05). The overall model fit meets the standard criteria, indicating a good fit.

The results indicate that avatar identification statistical significance influences immersion (β=.961; *P*<.001). Immersion statistical significance affects game attractiveness (β=1.229; *P*<.001). However, avatar identification does not directly affect game attractiveness (β=−.235; *P*=.28). This suggests that immersion fully mediates impact in the relationship, as shown in Table 8.

**Table 8:** Immersion fully mediates impact—mediation effect analysis.

Pathway	Unstandardized coefficient estimate	Standardized coefficient estimate	CR^a^	*P* value
VAI^b^ to DI^c^	1.041	0.961	17.333	<.001
DI to GA^d^	1.090	1.229	5.388	<.001
VAI to GA	–0.226	−0.235	–1.082	.28

^a^CR: critical ratio.

^b^VAI: virtual avatar identity.

^c^DI: immersion.

^d^GA: game attractiveness.

In the hypothesis testing of the mediating effect, the total mediating effect was significant (*P*<.001). Regarding the direct impact, both the effect of avatar identification on immersion and the effect of immersion on game attractiveness were significant (*P*<.001). However, the direct effect of avatar identification on game attractiveness was not significant (*P*=.28). Regarding the indirect impact, the effect of avatar identification on game attractiveness was significant (*P*<.001), as shown in Table 9.

**Table 9:** Total effects (*P*<.001).

Mediation pathway	Total effects			Direct effects				Indirect effects			
	*P* value	Bootstrapping		*P* value	Bootstrapping			*P* value		Bootstrapping	
		Bias corrected	Percentile		Bias corrected	Percentile			Bias corrected		Percentile
VAI^a^ to DI^b^	<.001	0.924 to 1.168	0.930 to 0.982	<.001	0.924 to 1.168	0.930 to 0.982	<.001		0.000 to 0.000		0.000 to 0.000
DI to GA^c^	<.001	0.709 to 1.745	0.827 to 1.969	<.001	0.709 to 1.745	0.827 to 1.969	<.001		0.000 to 0.000		0.000 to 0.000
VAI to GA	<.001	0.789 to 1.035	0.889 to 0.978	.28	−0.919 to 0.162	−0.975 to 0.170	<.001		0.748 to 1.973		0.799 to 1.943

^a^VAI: virtual avatar identity.

^b^DI: immersion.

^c^GA: game attractiveness.

**Figure 6:**
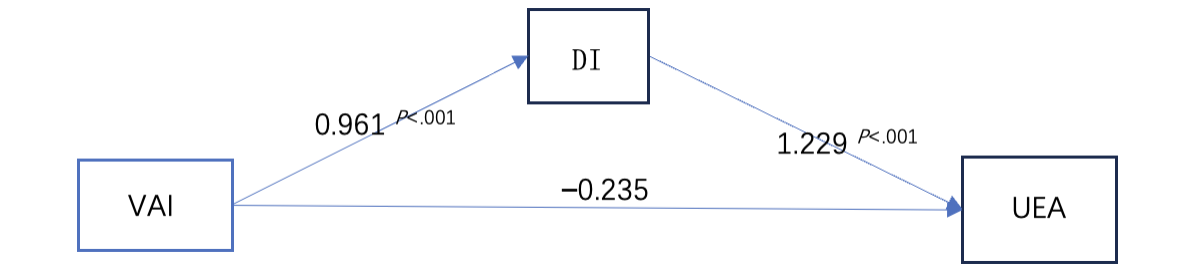
Immersion plays a fully mediating role—results of the validation of the mediation hypothesis model. DI: immersion; UEA: user experience attractiveness; VAI: virtual avatar identity.

On the basis of the hypothesis-testing analysis of the model, a hypothesis mediation model was obtained, as shown in Figure 6. Immersion fully mediates the effect of avatar identification on game attractiveness.

## Discussion

### Principal Findings

This study on avatar identification and game attractiveness discussed the relationship between players’ avatar identification and the attractiveness of VR games. It argued that immersion plays an important mediating role, helping VR game players establish a sense of identity with their virtual avatars. This study hypothesized that the implementation of avatar identification in VR games was positively influenced by dimensions of self-differentiation. The results show that immersion fully mediates the relationship between avatar identification and the attractiveness of the game to players. In addition, factors such as the similarity to the real self, the disparity between the real and ideal selves, the ideal self, and social behavior statistically significantly influence avatar identification. Moreover, avatar identification does not have a direct significant positive effect on game attractiveness, which aligns with our expectations for this study.

### Impact of Avatar Self Similarity, the Difference Between the Real and Ideal Selves, the Ideal Self, and Social Behavior on Identification With Avatars

The results of this study indicate that similarity to the authentic self was a significant positive predictor of identification with avatars; thus, hypothesis 1.1 was confirmed. In particular, similarity in the physical characteristics of the self positively impacted identification with avatars. Individuals choose avatars with traits similar to those of their authentic selves to achieve identification. Factors such as an individual’s abilities and personality in the real world also positively impact identification with avatars. This is consistent with previous conclusions that self-identity similarity, interaction similarity, and emotional similarity are positively correlated with avatar identification [24-26]. The difference between the authentic and ideal selves was a significant positive predictor of identification with avatars, confirming hypothesis 1.2. In turn, this confirms that motivational factors such as personal achievement, self-worth realization, virtual socialization, and transcending the authentic self positively impact identification with avatars, consistent with self-discrepancy theory [9] and demonstrating the proactive role of players in gaming behavior [27]. The ideal self had a significant positive predictive effect on identification with avatars, thus confirming hypothesis 1.3. This result shows that goal and cultural identification are positive aspects of the ideal self. Players’ attitudes toward narrative plots, cultural scenes, and artistic expressions and the degree of affection for individual characters determine the degree of identification with avatars. This is consistent with previous conclusions that ideal identification is formed in the construction of subjective identity [28,29]. Social behavior was a significant positive predictor of identification with avatars, which validates hypothesis 1.4. This result confirms that players’ value judgments and the achievement of meaning are determinants of their identification with social groups. The simulated interactive behaviors of players in the game and the investment of time, money, and energy in the game have a significant positive impact on identification with avatars. This is consistent with previous research conclusions [30].

### Impact of Identification With Avatars on Immersion

The results of this study show that identification with avatars is a significant positive predictor of immersion; thus, hypothesis 2 was confirmed. This research focused on the unique embodied sense of immersion that VR provides as the core of players’ gaming experience. To address this issue, immersion was divided into 3 types: perceptual, emotional, and behavioral. The findings confirm that avatars positively affect self-examination of audiovisual sensations, psychological feelings, and simulated interactive experiences (perceptual, emotional, and behavioral immersion, respectively). This is consistent with the conclusion by Suler [36] that technology, the environment, and the physical body are highly integrated in intrusive interactive environments, impacting cognition, behavior, and lifestyle [35-38].

### The Mediating Role of Immersion in the Relationship Between Identification With Avatars and Game Attractiveness

The results of this study indicate that immersion fully mediates the relationship between identification with avatars and game attractiveness to players. Therefore, hypothesis 3 was essentially verified; immersion plays a fully mediate effect, supporting the findings of previous research [39]. The presence of mediation suggests that immersion plays a significant role in influencing game attractiveness. The impact of avatar identification on game attractiveness only occurs positively when mediated by immersion. Immersion refers to total involvement in an activity, resulting in fulfillment and happiness.

On the basis of this survey, it was found that there is a clear positive correlation between avatar identification and immersion and immersion had a completely mediating effect on the relationship between avatar identification and VR game attractiveness. This finding will further prove the important role of avatar identification factors in generating user interest in VR games. Previous research has focused more on the comparative differences between immersion and presence, suggesting that VR games have more presence and gain game appeal compared to traditional video games. Therefore, in this study, immersion still played a significant role in mediating the relationship between avatar identification and VR game attractiveness.

### The Impact of Avatar Identification on Game Attractiveness

Our research showed that avatar identification does not have a direct positive correlation with game attractiveness; instead, avatar identification can only influence game attractiveness through immersion. Thus, hypothesis 4 was not supported in this study.

### Limitations

This study has certain limitations. First, the sample of participants in the survey was relatively small, which resulted in the inability to obtain a wider range of data. However, the data from this sample size are completely consistent with the study’s hypothesis, which to some extent supports the inferred results. Second, this study only explored the independent gaming experience of individual users without considering the social relationships between individuals and others, as well as the influence of personal and environmental space, which may be related to avatar identification and immersion. However, these elements require a combination of quantitative and qualitative experimental methods, which was not suitable for the questionnaire survey in this experiment. However, this study is already a very complete experimental process to address users’ self-identity with the gaming experience, and the experimental results will also have important reference value for further research on the impact of avatar identification on individual social space construction.

### Further Research

This study had limitations in investigating the relationship among avatar identification, social relationships, and spatial environments. Many factors could be explored in the context of whether and how a player’s perceived self, social relationships in the real world, and relationship with the actual physical space influence the individual avatar, the avatar’s interactions with NPCs and other players, and the spatial narrative of the game. We hope that this work promotes deeper theoretical reflection and empirical research in subsequent studies.

Moreover, the study design did not allow for rigorous causal inference. Due to time and cost constraints, conducting more detailed one-on-one interviews involving a larger sample was impossible, which may limit the richness of the information we obtained.

### Conclusions

In a future in which VR games are increasingly commonplace, avatars will serve as an identity tag for everyone. Therefore, from the perspective of VR games, this study investigated whether the disparity between the authentic and ideal selves of players affects identification with avatars and whether immersion has a mediating role in the relationship between avatar identification and the game’s attractiveness to players. The goal was to guide content creation for VR games to enhance players’ willingness to consume these games. This research used the SOR psychological paradigm as the model framework, with avatar identification as the stimulus element, immersion as the organism element, and the game’s attractiveness to players as the response element. Quantitative research methods were used. In conclusion, similarity to the authentic self, the difference between the real and ideal selves, the ideal self, and social behavior statistically significantly positively impacted identification with avatars. Immersion fully mediated the relationship between avatar identification and the game’s attractiveness to players, and avatar identification statistically significantly positively impacted game attractiveness.

This study explored methods to enhance the attractiveness of VR games by examining the relationship between avatar identification and immersion. This research showed that real self similarity, the discrepancy between the real and ideal selves, the ideal self, and social behavioral identity statistically significantly affect avatar identification, whereas immersion plays a complete mediating role between avatar identification and user experience attractiveness.

This research will benefit VR game development as it guides how game creators can better understand the concept of avatar identification. It suggests considering players’ physiological and psychological characteristics to understand their relationships with in-game characters. It also encourages developers to strive for proactive engagement with game content based on players’ gaming motivations and their identification with goals, environments, and cultures. Furthermore, this study offers rational guidance for designing player-friendly interaction modalities based on player behavior. In addition, by focusing on the roles of avatar identification and immersion, VR game designers should be able to enhance their games’ attractiveness, ultimately increasing gamers’ willingness to consume them. In the future digital era, avatar identification and immersion will not only influence the attractiveness of VR games but also impact people’s daily lives in the digital world. This raises the question of how to assist digital space designers in better enabling people to live in the metaverse’s digital space.

## Abbreviations

NPC: nonplayer character
SOR: stimulus-organism-response
VR: virtual reality

## Data Availability

The datasets generated during and analyzed during this study are available from the corresponding author on reasonable request.
